# Factors influencing the use of microbiology services: A cross-sectional study in Ethiopian public hospitals

**DOI:** 10.1186/s41043-025-01081-0

**Published:** 2025-09-30

**Authors:** Kibrewossen Kiflu Akililu, Workagegnehu Tarekegn, Zerihun Shimelis Kasa, Michael Solomon Tessema, Biniyam Tedla Mamo, Yemane Berhane

**Affiliations:** 1 Addis Continental Institute of Public Health, Addis Ababa, Ethiopia; 2 Ohio State Global One Health, Addis Ababa, Ethiopia; 3 Department of Nutrition and Behavioral Sciences, Addis Continental Institute of Public Health, Addis Ababa, Ethiopia; 4 Infection Prevention and Control Unit, Zewditu Memorial Hospital, Addis Ababa, Ethiopia; 5 Department of Epidemiology and Biostatistics, Addis Continental Institute of Public Health, Addis Ababa, Ethiopia

**Keywords:** Microbiology services, Ethiopia, Diagnostic stewardship, Limited resource setting, Microbiology in clinical practice, Antimicrobial resistance, Antimicrobial stewardship

## Abstract

**Background:**

Empiric management of infectious diseases is prevalent in low-resource settings. This has resulted in the emergence and spread of antimicrobial resistance. In Ethiopia, there is scarce evidence on the extent of use and factors influencing the utilization of microbiologic services in routine clinical practice. This study aims to address this gap.

**Methods:**

A cross-sectional study was conducted on 400 clinicians from eight public hospitals in Addis Ababa, Ethiopia. After obtaining informed consent, participants completed a self-administered questionnaire. The collected data was cleaned and analyzed using SPSS 27, with binomial and multinomial regression tests performed to measure statistical association between identified factors and microbiologic service utilization.

**Result:**

Two-hundred-twelve (53.0%) of the 400 clinicians had limited knowledge on the availability and/or types of microbiologic services provided in their facilities. Only fifty-nine (14.8%) consistently sent out culture tests when clinically indicated. The primary reasons for clinicians’ reluctance to prescribe culture tests included perceived gaps in availability, turnaround time, completeness of antimicrobial susceptibility test (AST) panels, and lack of concordance with clinical pictures. Besides their overall impact on diagnostic stewardship, these gaps have also left a negative impression on prescribers. Discordance with clinical profile (AOR = 0.28, 95% CI = 0.09, 0.88, *P* = 0.03), and incomplete AST panels (AOR = 0.82, 95% CI = 0.11, 0.94, *P* = 0.04) have negatively affected the perception interviewed clinicians had on reliability of services provided.

**Conclusion:**

The findings highlight key areas for targeted intervention in the knowledge, attitudes, and use of microbiology services in public healthcare, largely due to concerns about service quality and timeliness. The determinant factors identified present opportunities to improve laboratory services and better support clinicians in their practice. Despite the critical role of culture and sensitivity tests in combating antimicrobial resistance (AMR), their use remains limited. This underscores the urgent need for coordinated action to strengthen microbiology services as a key strategy in the fight against AMR.

**Table:** 

**Text box 1. Contributions to the literature**
• There is a significant paucity of data addressing microbiologic evidence-based management of infectious pathologies in clinical practice.
• Microbiologic data is the critical element in the rational use of antimicrobials, early detection of emerging multidrug resistant pathogens and targeted infection control measures.
• Proper utilization of microbiologic data is instrumental in the fight against antimicrobial resistance and needs to be given adequate attention from relevant stakeholders, policy makers and the international scientific community.
• Programmatic interventions combating antimicrobial resistance globally should place adequate emphasis on microbiologic evidence-based clinical practice.

## Introduction

Evidence-based clinical practice is inadequately implemented in low and middle-income countries (LMICs) such as Ethiopia [1, 2]. Empirical management of infections is also prevalent in settings with limited access to reliable microbiology services [3]. This issue is particularly pronounced in public facilities where resources are most scarce. Poor diagnostic stewardship has led to the over-prescription and mis-prescription of antimicrobials and emergence of antimicrobial resistance [3]. Several factors have contributed to the limited role of microbiological evidence in routine clinical practice in LMIC. Questionable quality of results, poor correlation with patients’ condition, and frequent interruption of services are some of the most important ones. However, this issue has not been addressed in any Ethiopian study.

In Africa, lack of access to reliable diagnostic tests has placed a negative impact on precision of clinical diagnosis and physicians’ trust on laboratory results [4]. A 2019 study from Nigeria, specifically addressed the extent and reasons for the underutilization of microbiology services. In this study, 65.8% of participants “often” ordered microbiological tests before treating infections. However, delayed results and uncertainty in the quality of results were cited by those who were reluctant to follow an evidence based approach [5]. Though not specific to microbiology services, a study conducted on 60 public hospitals in Ethiopia has shown that 71% of physicians had concerns on the accuracy of lab results [6]. These evidences indicate that clinicians’ perception on the quality of lab services affects their utilization.

Antimicrobial resistance (AMR) is one of the most concerning and emerging global threats to humanity [7, 8]. It was labeled as one of the top ten global health threats by World Health Organization (WHO) in 2021 [9, 10, 11]. AMR directly or indirectly has contributed to nearly 5 million deaths globally with low and middle income countries shouldering the major burden of its impacts [12]. The death toll from AMR alone is projected to reach 10 million annually by 2050, excluding its broader impacts. This calls for a stronger and more effective approaches in the coming years.

Efforts to curb the emergence and spread of drug resistant pathogens are mainly framed in three categories: antimicrobial stewardship, early detection and containment of multidrug resistant pathogens [13]. Rational use of antimicrobials is mainly achieved through diagnostic and therapeutic stewardship [14]. Implementing infection control also requires the early detection of multidrug-resistant pathogens circulating within healthcare facilities, highlighting the crucial role microbiological evidences in the fight against AMR.

In Ethiopia, where healthcare is underfunded, AMR is well prevalent. According to a systematic review from 14 studies, the pooled proportion of extended spectrum beta-lactamase-producing gram-negative bacteria was 50% in Ethiopia [15]. Another Ethiopian study highlighted the heavy reliance on clinical evidences while prescribing antimicrobials. In this study, 96.2% patients received empiric antimicrobial therapy [16]. Without consistent, high-quality microbiology support, clinicians lack the confidence to base treatment on lab results, underlying the need for national strategies that integrate diagnostic strengthening with antimicrobial stewardship.

This critical gap in diagnostic and therapeutic stewardship is a pressing issue in a nation with a high burden of AMR. Addressing the gap in clinical practice requires situational analysis and better understanding of the underlying factors. To date, no study has assessed the extent and factors influencing the use of microbiology lab services in Ethiopia. This study aims to fill that information gap focusing on bacteriologic antimicrobial susceptibility testing (AST). AST was given mere emphasis as it is the most informative microbiologic test for clinical use, IPC interventions and AMR surveillance.

## Methods

This cross-sectional hospital-based study was conducted from January-March, 2024 in eight public hospitals in Addis Ababa, Ethiopia, focusing on service utilization and the determinant factors from clinicians’ perspectives. Addis Ababa has 14 public hospitals serving an estimated population of 6 million and operating as referral centers for health facilities across the nation. These hospitals employ clinicians at various levels of profession and under different clinical departments. Five departments (Emergency Medicine, Internal Medicine, Surgery, Obstetrics and Gynecology (OBGYN), and Pediatrics) are designated as essential and a larger portion of the health task force is deployed under these clinical units.

The study’s source population included clinicians from 8 of the 14 public hospitals in Addis Ababa that provide bacteriology AST services. The clinician population comprised interns (final-year undergraduate medical students), health officers, general practitioners, residents (graduate physicians in specialty programs), specialists, fellows (senior specialists subspecializing in an area of expertise), and subspecialists.

### Sample size

A number of factors can potentially influence clinicians’ utilization of microbiologic data. While result quality, concordance with clinical picture and turnaround time were mentioned by literatures discussed in the background additional factors including awareness on service availability, cost, service interruption and completeness of AST test panels were considered in this study. The determinant role of these factors was assessed based on the accounts of clinicians.

According to the Ethiopian study discussed under background [6], 71% of physicians expressed concerns on the quality of lab services/results. However, no studies have assessed clinicians’ perception on the other potentially influencing factors. Thus, a 50% perception prevalence rate was estimated for the remaining factors to maximize the study’s power. The sample size for each parameter was calculated using a single proportion formula for a cross-sectional study. A sample size of 307 obtained for perception on quality (71%) while it was 384 for all other factors whose perception prevalence was unknown (estimated 50%).

Taking the larger of the two sample sizes, a size of 384 was obtained. Considering a 10% non-response rate, a sample size of 427 clinicians was set. A sample size of 54 was assigned to eight study facilities and participants were offered to fill out a self-administered questionnaire to assess their practice of utilization of microbiology services particularly culture and sensitivity tests. The questionnaire was offered both in paper and digital forms [Google form] and participants were allowed to choose.

### Data management and data quality control

The questionnaire was initially piloted in digital form at one of the study sites. Based on identified gaps, both digital and paper forms were subsequently modified. Most notably, selection of multiple choices was allowed where required. Data collectors were recruited (one per facility) and trained on the questionnaire’s content and administration procedures. Study participants on the other hand were given detailed orientation on the objectives, contents and administration techniques of the questionnaire before enrollment. For paper-based questionnaires, data collectors assessed completeness on the spot and kindly requested participants to fill in any missing values. In the Google Form, questions were set as “required” to ensure completeness. Utmost care was taken to avoid bias. Anonymity was ascertained and questions were kept simple and clear. Caution was taken to avoid double-barreled and leading questions.

### Data analysis procedures

Data collected via paper forms were entered into the Google Form after their alignment with digitally collected data was ascertained. Data was later downloaded in excel for further cleaning and then exported to SPSS version 27 for analysis. Analysis included simple descriptive ones. Bivariable analysis was conducted between several of the studied variables and dependent variables with dichotomous outcomes (reliability of lab result) using binomial regression while multinomial regression was used for polytomous ones (service utilization practice). A *P* value of 0.25 was set to select variables with significant association during bivariable analysis. Those with *P* < 0.25 were selected for multivariable analysis during which a cut off *P* value of 0.05 was used to determine association. Fitness test was conducted and collinearity between variables assessed with variance inflation factor.

### Ethical consideration

Ethical clearance was obtained from Addis Continental Institute of Public Health Ethical Review committee and Addis Ababa Health Bureau ethical board. Participants were advised on their right to or not to take part in the study and their right for refusal was respected. They were not incentivized in any way. Questionnaires were self-administered to avoid bias and deidentified to ensure confidentiality of the information obtained.

## Result

### Baseline characteristics of clinicians

A total of 400 clinicians from the eight public hospitals were willing to fill out the self-administered questionnaire. Except for one hospital where only 28 clinicians participated, in all facilities a minimum of 50 physicians took part in the study. The mean and median of age of participants was 31 and 30 respectively. 41% (163/400) were females and 59% (237/400) males. One hundred sixty (40%) were general practitioners (GPs) and 20% (80) were specialist clinicians (20%) by profession. Their year of experience ranged from three months for interns to 47 years for subspecialist physicians with the median year of experience of 5 years. Majority (55%, 228/400) worked in both inpatient and outpatient clinical service areas and one third (34%, 135/400) were working under Internal Medicine department. (See Tables 1, 2 and 3 below).

**Table 1:** Baseline characteristics of clinicians per facility

Variable	Outcomes	TASH *N* (%)	SPH *N* (%)	St Peter *N* (%)	ALERT *N* (%)	ZMH *N* (%)	Menelik *N* (%)	Yek12 *N* (%)	Ab-Gob *N* (%)	Tot *N* (%)
**Age**	**Range**	24–47	27–56	24–58	27–40	27–54	24–41	24–45	23–41	23–58
	**Mean**	31.02	32.75	31.55	32.74	33.53	31.07	30.46	28.57	31.35
	**Median**	30	32	30	33	32	30	30	28	30
**Sex**	**Male**	30 (58.8%)	38 (71.7)	34 (64.2)	11(40.7)	30 (58.8)	25 (43.9)	34 (65.4)	35 (62.5)	237 (59.3)
	**Female**	21 (41.2%)	15 (28.3)	19 (35.8)	16 (59.3)	21 (41.2)	32 (56.1)	18 (34.6)	21 (37.5)	163 (40.8)
**Total**		51 (12.8)	53 (13.3)	53 (13.3)	27 (6.8)	51 (12.8)	57 (14.2)	52 (13)	56 (14)	400 (100)

TASH-Tikur Anbessa Specialized Hospital, SPH-St Paul’s Hospital, ZMH-Zewditu Memorial Hospital, Yek12-Yekatit 12, Ab-Gob-Abebech Gobena

**Table 2:** Professional stand and experience of clinicians per each facility

Variable	Outcomes	TASH *N* (%)	SPH *N* (%)	St Peter *N* (%)	ALERT *N* (%)	ZMH *N* (%)	Menelik *N* (%)	Yek12 *N* (%)	Ab-Gob *N* (%)	Tot *N* (%)
**Year of experience**	**Range**	0.25-15	2–25	0.25-47	0.25-15	2–27	0.25-12	0.25-11	0.2-7	0.25-47
	**Mean**	4.82	7.72	6.47	6.25	8.34	4.72	3.68	2.24	5.45
	**Median**	4	6	5	6	7	4	3	1	5
**Level of profession**	**Health officer**	0	0	1 (1.9)	1 (3.7)	0	0	0	0	2 (0.5)
	**Intern**	5 (9.8)	0	6 (11.3)	2 (7.4)	0	12 (21.1)	12 (23.1)	23 (41.1)	60 (15)
	**General Practitioner**	3 (5.9)	5 (9.4)	34 (64.2)	14 (51.9)	28 (54.9)	34 (59.6)	25 (48.1)	17 (30.4)	160 (40)
	**Resident**	25 (49.0)	14 (26.4)	6 (11.3)	0	0	6 (10.5)	12 (23.1)	14 (25.0)	77 (19.3)
	**Specialists**	16 (31.4)	24 (45.3)	3 (5.7)	9 (33.3)	19 (37.3)	4 (7)	3 (5.8)	2 (3.6)	80 (20)
	**Sub-specialists**	2 (3.9)	9 (17)	2 (3.8)	1 (3.7)	4 (7.8)	1 (1.8)	0	0	19 (4.8)
**Total per facility**		51 (12.8)	53 (13.3)	53 (13.3)	27 (6.8)	51 (12.8)	57 (14.2)	52 (13)	56 (14)	400 (100)

TASH-Tikur Anbessa Specialized Hospital, SPH-St Paul’s Hospital, ZMH-Zewditu Memorial Hospital, Yek12-Yekatit 12, Ab-Gob-Abebech Gobena

**Table 3:** Clinicians’ site of service delivery per each facility

Variable	Outcomes	TASH *N* (%)	SPH *N* (%)	St Peter *N* (%)	ALERT *N* (%)	ZMH *N* (%)	Menelik *N* (%)	Yek12 *N* (%)	Ab-Gob *N* (%)	Tot *N* (%)
**Department**	**Int-Med**	10 (19.6)	20 (37.7)	23 (43.4)	10 (37)	24 (47.1)	16 (28.1)	31 (59.6)	1 (1.8)	135 (33.8)
	**Surgery**	9 (17.6)	28 (52.8)	5 (9.4)	0	3 (5.9)	8 (14)	3 (5.8)	2 (3.6)	58 (14.5)
	**Pediatrics**	6 (11.8)	1 (1.9)	10(18.9)	8 (29.6)	7 (13.7)	21 (36.8)	5 (9.6)	10 (17.9)	68 (17)
	**OBGYN**	2 (3.9)	0	0	0	7 (13.7)	1 (1.8)	1 (1.9)	42 (75)	53 (13.3)
	**ER Medicine**	9 (17.6)	3 (5.7)	10(18.9)	0	7 (13.7)	9 (15.8)	2 (3.8)	1 (1.8)	41 (10.3)
	**Others**	15 (29.4)	1 (1.9)	3(5.66)	9 (33.3)	3 (5.9)	2 (3.5)	7 (13.5)	0	40 (10)
	**UK**	0	0	2(3.8)	0	0	0	3 (5.8)	0	5 (1.3)
**Site of service delivery**	**OPD**	1 (2)	0	7 (13.2)	2 (7.4)	9 (17.6)	6 (10.5)	6 (11.5)	1 (1.8)	32 (8)
	**IPD**	4 (7.8)	1 (1.9)	13 (24.5)	5 (18.5)	4 (7.8)	17 (29.8)	6 (11.5)	1 0(17.9)	60 (15)
	**ER**	6 (11.8)	0	17 (32.1)	0	8 (15.7)	8 (14)	4 (7.7)	1 (1.8)	44 (11)
	**ICU**	14 (27.5)	0	6 (11.3)	1 (3.7)	3 (5.9)	9 (15.8)	0	1 (1.8)	34 (8.5)
	**OPD & IPD**	19 (37.3)	44 (83)	8 (15.1)	16 (59.3)	22 (43.1)	15 (26.3)	30 (57.7)	39 (69.6)	193 (48.3)
	**All**	7(13.7)	8(15.1)	1 (1.9)	3()	4(11.1)	2(3.5)	6(11.5)	4(7.1)	35(8.8)
**Total per facility**		51 (12.8)	53 (13.3)	53 (13.3)	27 (6.8)	51 (12.8)	57 (14.2)	52 (13)	56 (14)	400 (100)

TASH-Tikur Anbessa Specialized Hospital, SPH-St Paul’s Hospital, ZMH-Zewditu Memorial Hospital, Yek12-Yekatit 12, Ab-Gob-Abebech Gobena

### Clinicians knowledge on service availability

**Fig. 1:**
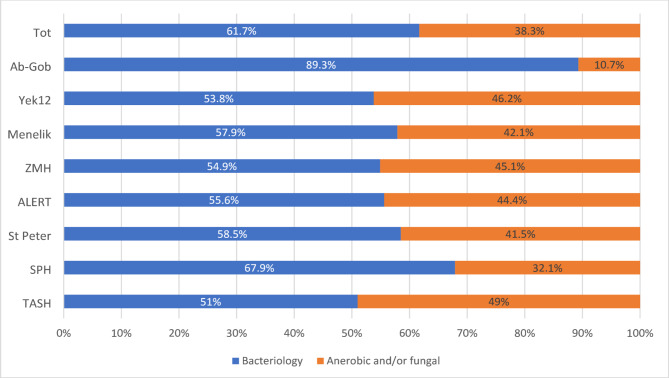
Clinicians knowledge on type service available. TASH-Tikur Anbessa Specialized Hospital, SPH-St Paul’s Hospital, ZMH-Zewditu Memorial Hospital, Yek12-Yekatit 12, Ab-Gob-Abebech Gobena

**Fig. 2:**
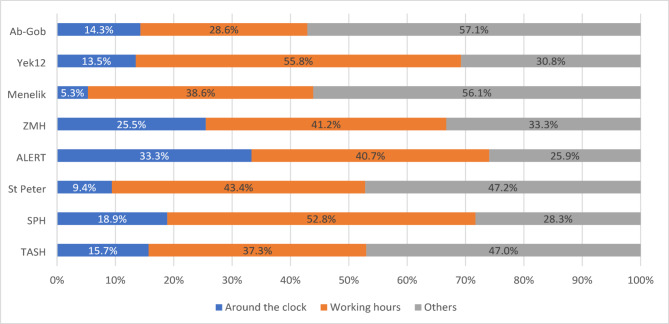
Clinicians knowledge on time of service availability. TASH-Tikur Anbessa Specialized Hospital, SPH-St Paul’s Hospital, ZMH-Zewditu Memorial Hospital, Yek12-Yekatit 12, Ab-Gob-Abebech obena

Out of 400 clinicians, 6.5% [26] believed their facilities lacked microbiology services while 8.25% (33) were unaware of the type of culture and sensitivity tests offered. 38.3% (153/400) believed their facility’s labs conducted fungal and/or anerobic bacterial culture, services none of the eight labs were delivering during the study period. Moreover only 16% (63/400) were aware microbiology laboratory worked around the clock. In general, a palpable information gap was observed from studied participants. (See Figs. 1 and 2 below)

### Utilization of culture and sensitivity services and influencing factors

344 of the 400 physicians (86%) were involved in the clinical care of patients with infections for at least 1–3 times a week. However only 59(14.8%) sent out culture tests consistently when clinically indicated (suspected local or systemic infections). Four (1%) preferred empiric clinical management to basing antimicrobial therapy on culture results (Tables 4 and 5).

Several factors were shown to influence clinicians’ practice of sending culture specimens.

- 39% felt facility-based culture services were not consistently available,
- 23% felt results arrived too late for meaningful clinical use,
- 14% believed AST test panels were not comprehensive enough for patients’ clinical needs,
- 12% felt results issued did not reflect the clinical picture of their patients and.
- 7% had cost concerns.

Overall, 13% felt the culture services provided by their facilities’ labs did not provide clinically impactful results.

**Table 4:** Clinicians’ frequency of encounter with patients with infectious diseases

Variable	Outcome	TASH	SPH	St Peter	ALERT	ZMH	Menelik	Yek12	Ab-Gob	Tot
**Encounter with patients with infection**	Daily	34 (66.7%)	23 (43.4%)	28 (52.8%)	14 (51.9%)	30(58.8%)	29(50.9%)	39(75%)	19(33.9%)	216 (54%)
	4-6x/wk	8(15.7%)	18(34%)	1 (1.9%)	3(11.1%)	8(15.7%)	9(15.8%)	3(5.8%)	13(23.2%)	63 (15.8%)
	1-3X/wk	4 (7.8%)	9(17%)	9(17%)	7(25.9%)	4(7.8%)	14(24.6%)	7(13.5%)	11(19.6%)	65(16.3%)
	1-3X/Mo	5(9.8%)	3(5.7%)	12(22.6%)	2(7.4%)	7(13.7%)	5(8.8%)	3(5.8%)	8(14.3%)	45(15.3%)
	1-10d/yr	0	0	2(3.8%)	1(3.7%)	2(3.9%)	0	0	4(7.1%)	9(2.3%)
	Never	0	0	1(1.9%)	0	0	0	0	1(1.8%)	23(0.5%)
**Total**		51 (12.8)	53 (13.3)	53 (13.3)	27 (6.8)	51 (12.8)	57 (14.2)	52 (13)	56 (14)	400 (100)

TASH-Tikur Anbessa Specialized Hospital, SPH-St Paul’s Hospital, ZMH-Zewditu Memorial Hospital, Yek12-Yekatit 12, Ab-Gob-Abebech Gobena

**Table 5:** Frequency of use of culture tests in clinical practice

Variable	Outcome	TASH	SPH	St Peter	ALERT	ZMH	Menelik	Yek12	Ab-Gob	Tot
**Practice of sending cultures**	Always	13(25.5%)	7(13.2%)	7(13.2%)	3(11.1%)	9(17.6%)	8(14%)	2(3.8%)	10(17.9%)	59(14.8%)
	75–90%	13(15.5%)	4(7.5%)	12(22.6%)	4(14.8%)	6(11.8%)	12(21.1%)	4(7.7%)	10(17.9%)	65(16.3%)
	50–75%	7(13.7%)	16(30.2%)	8(15.1%)	7(25.9%)	6(11.8%)	17(29.6%)	16(30.8%)	9(16.1%)	86(21.5%)
	25–50%	11(21.6%)	18(34%)	9(17%)	7(25.9%)	8(15.7%)	12(21.1%)	14 (26.9%)	7(12.5%)	86(21.5%)
	< 25%	7(13.7%)	8(15.1%)	15(28.3%)	6(22.2%)	20(30.2%)	8(14%)	16(30.8%)	20(35.7%)	100(25%)
	Never	0	0	2(3.8%)	0	2	0	0	0	4(1%)
**Total**		51 (12.8)	53 (13.3)	53 (13.3)	27 (6.8)	51 (12.8)	57 (14.2)	52 (13)	56 (14)	400 (100)

TASH-Tikur Anbessa Specialized Hospital, SPH-St Paul’s Hospital, ZMH-Zewditu Memorial Hospital, Yek12-Yekatit 12, Ab-Gob-Abebech Gobena

Binary and multinomial regression tests were employed to determine the degree of association between influential factors and the outcomes of measure (the habit of sending cultures, perceived reliability of lab results). Accordingly, only gender, was strongly associated with the primary outcome of measure. Female physicians were 2.5 times more likely to send cultures for at least 50% of patients with clinical indications (AOR = 2.6, 95%CI = 2.05–4.74, *P* < 0.001). Table 6 shows the association between possible influential factors and the habit of sending culture tests by clinicians.

A similar analysis scheme was followed to further understand factors affecting clinicians’ perception on reliability of lab results. During bivariable analysis, clinicians’ profile, namely, deployment location, frequency of encountering infectious cases, their level of experience and professional standing were shown to have statistical association with perception on reliability. From lab’s side: result’s turnaround time, completeness of AST results and concordance with clinical picture had a notable association with perception on reliability. After adjusting for covariates, perception was only strongly associated with clinical concordance (AOR = 0.28, 95%CI = 0.09–0.88, *P* = 0.03), and completeness of test panels (AOR = 0.82, 95 CI = 0.11–0.94, *P* = 0.04). Clinicians who believed lab results well reflected patients’ clinical picture were 3.6 times likely to have a better perception on overall reliability of lab services. In addition, clinicians satisfied by variety of AST test panels had slightly better (1.2 times) positive impression.

**Table 6:** Association between possible influential factors and the habit of sending culture 50% of time

Variable	Outcome	COR	AOR	*P* value	95% CI
Age	-	1.01	1.02	0.60	0.97–1.05
Experience	-	0.99	0.95	0.47	0.95–1.03
Sex	Female	3.12	2.60	< 0.001	2.05–4.74
Knowledge on service availability	Yes	1.12	2.88	0.88	0.25–5.13
Service availability	Inconsistent	0.86	0.86	0.45	0.56–1.31
	Consistent				
Service cost	Expensive	0.76	0.94	0.52	0.33–1.75
	Fairly priced				
AST test results	Reliable	1.31	1.52	0.41	0.69–2.50
	Unreliable				
AST panel	Incomplete	0.86	0.82	0.63	0.47–1.57
	Complete				
Result delivery	Timely	1.51	1.48	0.10	0.92–2.47
	Late				
Profession	General Practitioners	1.08	-	0.70	0.07–17.53
	Health Officers	1.00	-	0.96	0.02–50.40
	Specialists	0.70	-	1.00	0.04–11.63
	Subspecialists	0.73	-	0.81	0.04–13.45
	Interns	0.71	-	0.83	0.04–11.97
	Residents	1.03	-	0.82	0.06–17.01
	Unknown	1.00	-	0.99	Ref
Department	Internal Medicine	1.52	-	0.01	0.25–9.40
	Surgery	2.28	-	0.65	0.35–14.74
	Pediatrics	0.50	-	0.39	0.08–3.25
	Obstetrics-Gynecology	1.81	-	0.47	0.28–11.75
	Plastics	1.50	-	0.53	0.18–12.46
	Anesthesiologists	3.00	-	0.71	0.37–24.17
	Emergency physician	0.67	-	0.30	0.08–5.68
	Other	1.30	-	0.71	0.20–8.589
	Unknown	0.67	-	0.79	Ref

## Discussion

It was interesting to note that 53% (212/400) of respondents were unaware of the presence or type of microbiology services in their facilities. In addition, only 16% knew services were being provided on a 24/7 basis. The study did not try to address the reasons for this information gap. However, the habit of sending cultures itself could possibly be an important reason, as clinicians would have been more acquainted with facility services the more they utilized them. Moreover, test menus in Ethiopian public health facilities are not updated and promoted on a regular basis. This can be another important factor for the knowledge gap noted. Unfortunately, awareness on lab services is not addressed well in similar other studies.

Practice of backing clinical decisions with microbiologic evidences was modest in all facilities. This was also reflected in a cross-sectional study conducted on 181 Ethiopian Health professionals in 2022. Eighty-one (44.8%) respondents did not base their chemotherapy on AST results [17]. However, use of culture tests reported in this study was significantly lower from what was reported in the Nigerian study (64%). The later yet had a smaller sample size (283) and simpler serologic and microscopy based microbiologic tests were also counted in [5].

Most importantly, lab utilization rate was paradoxically low compared to the frequency of encounter clinicians had with patients who required bacteriologic investigations. 86% (344/400) clinicians weekly evaluated minimum of 1–3 patients for whom culture and AST was indicated. This underscores the magnitude of malpractice that exists in terms of diagnostic stewardship.

Clinicians at TASH and St Paul reported higher prescription habits. These two are the two largest tertiary hospitals in Ethiopia and are teaching institutions. The academic environment may have promoted a better practice of diagnostic stewardship. On the contrary, the hierarchical culture in academic medical institutions was mentioned as one important reason for placing patients on more broad spectrum antimicrobials in a survey conducted at TASH [18]. Role of an academic environment in the implementation of diagnostic and antimicrobial stewardship is one gray area that calls for a closer look and an in-depth situational analysis. The practice of sending cultures was interestingly better among female clinicians. In two studies focused on management of patients with breast cancer, female physicians were noted to comply more to clinical guidelines as to their male counterparts [19, 20]. The assertive and risk aversion nature of female clinicians could be one reason for this variation.

The physicians under this study have indicated a number of factors hindering the routine clinical use of culture and sensitivity tests. Major gaps in consistency, timeliness, reliability, comprehensiveness and cost were mentioned by respondents. Moreover, 13% of clinicians did not believe microbiology services delivered at facility labs were clinically impactful. These can be strong reasons for prescribers’ hesitancy. The role of limited availability of quality diagnostic tools in poor stewardship practices is addressed in a position statement for the international society for infectious diseases [21]. This position statement also outlines several other physicians related and external factors influencing diagnostic stewardship. So did a systematic review discussing the reasons behind poor antimicrobial stewardships [22]. However, both of these articles fail short of addressing the laboratory element.

Time of result delivery was mentioned as one strong factor for a number of outcome variables. Yet, timeliness itself was significantly different among various facilities. This can be the effect of sample volume as those facilities where TAT was mentioned as an issue were noted to have higher adjusted sample volume. The clinicians from the previously mentioned Nigerian study mentioned delayed result delivery and unreliability as reasons to defer culture tests [5]. Reliance for general lab services was also mentioned as an important gap in an Ethiopian survey conducted by Hailu et al. which showed 71% clinicians had concerns about quality of results delivered by their labs [6]. In a health system where clinicians have doubts in quality of lab services, it would be quite natural to expect higher reliance on empiric management or tendency to refrain from use of available services.

One possible solution to address the lab utilization and reliance is improved lab-clinician communication. Effective communication between laboratory professionals and clinicians is essential to ensuring timely and clinically relevant patient care. It facilitates early delivery of critical and routine laboratory results, keeps clinicians informed about newly available test panels or changes in test offerings, and enables laboratories to tailor result interpretation to the patient’s specific clinical context. This improves the clinical utility, accuracy, and reliability of laboratory data, supporting better diagnostic and therapeutic decisions [23, 24]. When laboratory professionals are aware of the clinical background, they can provide more nuanced and context-driven interpretations especially important in complex or borderline results. Additionally, direct communication channels support the prompt reporting of critical values and reduce the risk of diagnostic errors due to misinterpretation or delayed follow-up [25].

Factors across the pre-analytic, analytic, and post-analytic phases of the laboratory testing process significantly influence clinicians’ use of and confidence in laboratory data. As described by Epner et al. in the concept of the ‘Total Testing Process’ and Lundberg’s Brain-to-Brain Loop, errors can occur at any stage from test selection and specimen collection to analysis, reporting, and interpretation. Pre-analytic issues such as poor sample handling or incorrect test orders are especially common and can undermine result accuracy. Similarly, analytic errors like equipment malfunction and post-analytic issues such as delayed reporting or misinterpretation) can reduce the clinical value of lab tests. Recognizing and addressing these potential obstacles is essential to improving lab utilization and ensuring reliable, patient-centered care [23, 26].

### Limitations and strengths of the study

This paper addresses the paucity of data that exists in the Ethiopian health care system with regards to microbiology services. The study was multicentered and its response rate was adequate enough to ensure power of the study. Moreover, several measures were taken to maintain quality of data. However, there were some drawbacks to its design. The study population was quite heterogenous across facilities and sample size was not stratified proportionally. Allocation of similar sample size to all eight study sites would have introduced a sample bias depriving the study participants from larger and academic institutions that potentially had better diagnostic clinical practices. Clustering adjustment was also not undertaken for correlated observations within hospitals.

The study gave emphasis for culture and sensitivity tests even though other microbiologic tests like gram stain, serologic tests, AFB stain and simple microscopy were the more widely available, frequently prescribed but fairly equally essential microbiologic tests in clinical practice. Even though, a more lenient and all-inclusive approach would have yielded better lab use results, we wanted to limit the scope to culture and AST as the other microbiological lab tests are both over and underutilized. That would have led to more confusing results. Moreover, the study on clinicians’ practice was completely dependent on the accounts of the clinicians themselves possibly introducing a reporting bias. This study also oversees other factors in labs’ capacity, performance and ways of operation that potentially play a significant role in determining use of lab services. A qualitative study with an in-depth situational analysis will be a reasonable way to address these factors.

## Conclusion and recommendation

This study highlights significant gaps in knowledge, attitude, and the use of facility-based microbiology services in public healthcare facilities. The quality and timeliness of these services strongly influenced clinicians’ reliance on microbiology services, indicating a need for targeted interventions and further research. Training programs addressing diagnostic and antimicrobial stewardship can enhance use and interpretation of AST. Additionally. recognition of better performing clinicians, units and facilities at large can serve as a strong motivator and promote best practices. Establishing a more effective communication platform between lab staff and clinicians can address gaps in awareness and concerns on lab services. It can also serve as a portal to receive feedbacks, deliver results timely and flag results that require urgent communication. Feedback systems between clinicians and lab staff will also build trust in either end, will promote lab utilization and improves clinical relevance of lab results.

The importance of evidence-based practice is of paramount importance, especially in the face of rising antimicrobial resistance. Culture and sensitivity tests play a crucial role by not only ensuring the appropriate use of antimicrobials but also providing detailed qualitative data on multidrug-resistant organisms and are important inputs for AMR surveillance at national level. However, the limited utilization of these services remains a significant challenge in health systems in LMICs. This highlights the pressing need for unified efforts among all stakeholders to strengthen and expand microbiology services nationwide, as their proper application is fundamental to any effective response to antimicrobial resistance.

## Data Availability

Most of the research data and its findings are presented in the manuscript. Additional raw data are available from the corresponding author upon reasonable request.

## Abbreviations

AMR: Antimicrobial resistance
GP: General practitioner
LMIC: Low- and middle-income countries
MDRO: Multi drug resistant organisms
OBGYN: Obstetrics and gynecology
PI: Primary investigator
SPH: St Paul’s hospital
SSH: Sub Saharan Africa
UN: United Nations
WHO: World health organization
TASH: Tikur anbessa specialized hospital
